# The effects of intermittent negative pressure on the lower extremities' peripheral circulation and wound healing in four patients with lower limb ischemia and hard‐to‐heal leg ulcers: a case report

**DOI:** 10.14814/phy2.12998

**Published:** 2016-10-26

**Authors:** Øyvind H. Sundby, Lars Ø. Høiseth, Iacob Mathiesen, Jørgen J. Jørgensen, Jon O. Sundhagen, Jonny Hisdal

**Affiliations:** ^1^ Section of Vascular InvestigationsDepartment of Vascular SurgeryDivision of Cardiovascular and Pulmonary DiseasesOslo University HospitalOsloNorway; ^2^ Faculty of MedicineInstitute of Clinical MedicineUniversity of OsloOsloNorway; ^3^ Otivio ASOsloNorway; ^4^ Department of AnesthesiologyDivision of Emergencies and Critical CareOslo University HospitalOsloNorway; ^5^ Department of Vascular SurgeryDivision of Cardiovascular and Pulmonary DiseasesOslo University HospitalOsloNorway

**Keywords:** Blood flow, intermittent negative pressure, leg ulcer, peripheral arterial disease, wound healing

## Abstract

Peripheral circulation is severely compromised in the advanced stages of peripheral arterial disease. Recently, it was shown that the application of −40 mmHg intermittent negative pressure (INP) to the lower leg and foot enhances macro‐ and microcirculation in healthy volunteers. In this case report, we describe the effects of INP treatment on four patients with lower limb ischemia and hard‐to‐heal leg and foot ulcers. We hypothesized that INP therapy may have beneficial hemodynamic and clinical effects in the patients. Four patients (age range: 61–79 years) with hard‐to‐heal leg and foot ulcers (6–24 months) and ankle‐brachial pressure indices of ≤0.60 on the affected side were included. They were treated with an 8‐week intervention period of −40 mmHg INP (10 sec negative pressure and 7 sec atmospheric pressure) on the lower limbs. A custom‐made vacuum chamber was used to apply INP to the affected lower leg and foot for 2 h per day. After 8 weeks of INP therapy, one ulcer healed completely, while the other three ulcers were almost completely healed. These cases suggest that INP may facilitate wound healing. The theoretical foundation is that INP assists wound healing by improving blood flow to the small blood vessels in the affected limb, increasing the flow of oxygen and nutrients to the cells.

## Background

Arterial leg ulcers result from insufficient blood supply to the tissues. In patients with advanced stages of peripheral arterial disease (PAD), ulcers of the lower leg are common, due to reduced microcirculation in the extremities (Mekkes et al. 2003; Grey et al. 2006). Unfortunately, the treatments available for these patients are often insufficient (Wolfe and Wyatt 1997), frequently causing complications and unnecessarily long healing times (Marston et al. 2006). Revascularization below the knee shows poor long‐term results (Gray et al. 2014). A common method used to treat acute and chronic wounds is topical negative pressure wound therapy (NPWT) where a pump applies continuous or intermittently negative pressure to a sealed dressing in the wound area, removing wound exudate from the exposed area into a canister (Dumville et al. 2016). Unfortunately, the use of NPWT in the local wound environment and its impact on healing of arterial leg ulcers and tissue perfusion in clinical use are inconclusive (Wackenfors et al. 2004; Gregor et al. 2008; Vig et al. 2011; Shon et al. 2014).

Recently, we developed a method that applies intermittent mild negative pressure (INP) to the lower leg and foot in a chamber sealed below the knee (Sundby et al. 2016). In a study on healthy volunteers, we observed increased acute arterial and skin blood flow in the foot (Sundby et al. 2016). The rationale for applying INP is the finding that constant negative pressure applied to an extremity causes venous distension and reduces blood flow locally via a vasoconstrictor mechanism, the veno‐arterial reflex (Skagen and Henriksen 1983). Smyth (1969) applied a similar INP methodology to the lower limb (−150 mmHg), and observed increased wound healing and walking distance after 6 weeks of INP‐therapy in patients with peripheral vascular disease (Smyth 1969). Despite these results, little attention has been given to the INP method in recent years.

Four patients with ischemic limbs and hard‐to‐heal leg and foot ulcers underwent 8 weeks of INP‐therapy (Table 1). In addition, we describe in detail two of the patients with the lowest foot perfusion at inclusion. The patients received standard wound care at the local hospital's wound clinic for 5–18 months before inclusion in this study. They agreed to take part in an 8‐week pilot study on the clinical effect of a novel INP method on wound healing. The experimental protocol was approved by the regional ethics committee (REK Sør‐Øst 2015/1318). Written informed consent was obtained from all patients to publish this report.

**Table 1:** Characteristics of the wound patients (*n* = 4) exposed to 8 weeks of INP‐therapy. See text for a detailed description of patient 1 and patient 2

Patients	Measurements	Demographic	Week 0 (Baseline)	Week 8 (Completion)
			Wound leg	Wound leg
1	Age (year)	63.0		
	Height (cm)	176.0		
	Weight (kg)	76.0		
	Time with wound (mo)	24		
	Wound size (cm)		6.5 × 2.7	4.5 × 1.8
	ABPI		0.50	0.67
2	Age (year)	61.0		
	Height (cm)	176.0		
	Weight (kg)	76.0		
	Time with wound (mo)	24		
	Wound size (cm)		4.0 × 4.5/1.6 × 1.6	1.5 × 1.5/Epithelialized
	ABPI		0.46	0.62
3	Age (year)	74.0		
	Height (cm)	180.2		
	Weight (kg)	75.7		
	Time with wound (mo)	6		
	Wound size (cm)		0.5 × 0.5	Epithelialized
	ABPI		0.51	0.54
4	Age (year)	79.0		
	Height (cm)	178.0		
	Weight (kg)	77.6		
	Time with wound (mo)	8		
	Wound size (cm)		4.5 × 3.5	Epithelialized
	ABPI^a^		N/A	N/A

ABPI, Ankle‐Brachial Pressure Index.

a Not applicable due to calcified vessels.

Based upon the aforementioned studies (Smyth 1969; Sundby et al. 2016), we hypothesized that INP‐therapy would increase macro‐ and microvascular capacity in the lower extremity, and that this would facilitate adequate perfusion for wound healing to occur. INP therapy was performed at home with a portable device (FlowOx^™^, Otivio AS, Oslo, Norway). The device consists of a vacuum chamber and a pump. The method is described in detail elsewhere (Sundby et al. 2016). The vacuum chamber is sealed around the patient's leg below the knee (Fig. 2). Negative pressure cycles are created by alternating between removing air (10 sec of −40 mmHg) and venting the chamber to atmospheric pressure (7 sec). The patients were instructed to use the INP device at home for a total of 2  h, divided into two sections of 1 h per day. Treatment compliance was observed by recording INP time on a USB memory stick provided with the device. Wound size for each patient was measured by the wound nurses prior to and after 8 weeks of INP‐therapy. Macrocirculation was assessed in a supine position by the ankle brachial pressure index (ABPI) with a hand‐held 8 MHz Ultrasound Doppler blood velocity detector. Additionally, a pulse volume recording (PVR) (MacroLab, STR Teknikk, Aalesund, Norway) was performed. Microcirculation was assessed by skin perfusion pressure (SPP) using SensiLase PAD‐IQ (Vasamed Inc., Eden Prairie, MN).

## Report

### Patient 1

The first patient was a 63‐year‐old Caucasian male, smoker, and non‐diabetic (Table 1). His medical history included myocardial infarction, a cerebral insult, smoking‐induced chronic obstructive pulmonary disease, paroxysmal atrial fibrillation, and a long‐standing history of leg ischemia with rest pain when sleeping. He had undergone several surgical procedures over the past few years, including a femoropopliteal bypass with a Propaten graft above the knee in his right leg 2 years before the INP‐treatment.

Within a few months following surgery, the patient experienced acute thrombosis of the graft and embolism distally in the leg arteries. Subsequently, a leg ulcer appeared on the patient's anterior tibia. The ulcer was treated unsuccessfully with a partial dermal skin graft from the patient's left thigh.

The patient regularly visited the wound clinic at the University Hospital to receive conventional wound care for 2 years prior to participating in the study. His maximal claudication distance was 100 m at a self‐selected pace due to severe pain in his right calf. The main concern was his poor arterial leg inflow. Upon vascular examination, the patient had intact femoral artery pulses bilaterally, but no pulses in the arteries of his right foot. Duplex color scanning revealed an occluded popliteal artery. Distally, the only open artery was the fibular artery, which was open in two‐thirds of the upper leg. The patient was informed that if the pain and wound worsened or failed to heal properly, amputation of the limb might be indicated.

**Figure 1:**
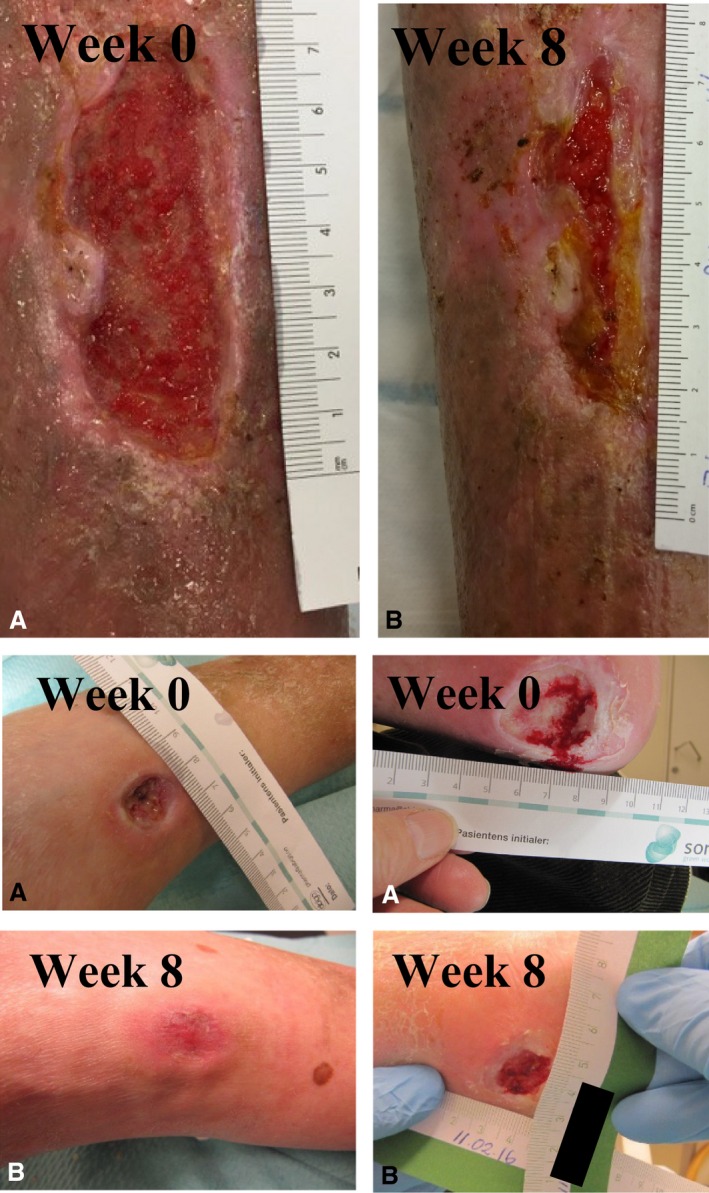
Upper panel: Picture of patient 1's leg ulcer before (A) and after (B) 8 weeks of INP therapy. Lower panel: Picture of patient 2's foot ulcers before (A) and after (B) 8 weeks of INP therapy. The pictures on the left are from the dorsum pedis (completely epithelialized after 8 weeks of INP therapy), and the pictures on the right are from the patient's heel (heel wound size after 8 weeks: 1.5 × 1.5 cm).

**Figure 2:**
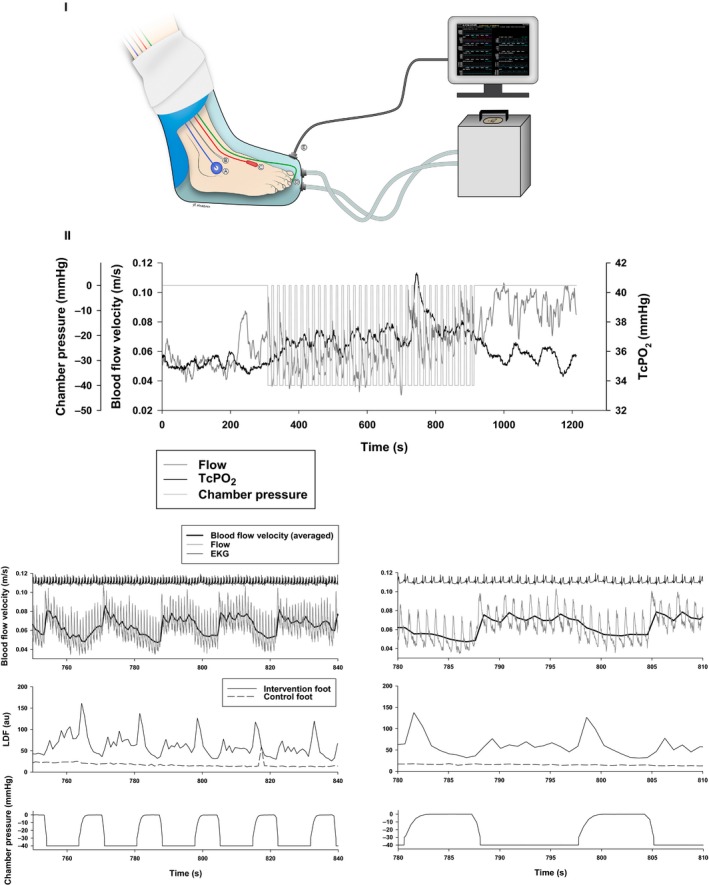
I: Illustration of the custom‐made airtight vacuum chamber and the INP generator used by the four wound patients. The illustration shows how the probes were attached to the foot when measuring arterial blood flow velocity, laser Doppler flux (LDF), and transcutaneous oxygen pressure (TcPO2) in the foot of patient 1 during INP‐therapy. (A) TcPO_2_ probe; (B) Skin temperature probe; (C) Ultrasound Doppler probe; (D) Laser Doppler flux probe; (E) The pressure transducer from the boot interfaced with the computer. Illustration: Øystein H. Horgmo, University of Oslo. II: Measures of acute hemodynamics in patient 1 after 8 weeks of INP. The upper large panel is TcPO_2_ and arterial blood flow in the dorsal pedis artery (flow velocity) during the whole 20‐min sampling period: 5 min atmospheric pressure, 10 min INP and 5 min atmospheric pressure. Lower six panels: Beat‐to‐beat measures during application of intermittent negative pressure (INP) in patient 1 zoomed in from 750 to 840 sec (left) and from 780 to 810 sec (right). The panels show blood flow velocity and pulp skin flow response in the patient's right foot during application of INP. Upper panels: Blood flow velocity (ultrasound Doppler – thin lines), averaged within heartbeats (thick lines). Middle panels: Laser Doppler flux. Lower panels: Chamber pressure during INP.

After 8 weeks of INP‐therapy 2  h per day, the leg ulcer almost healed (Fig. 1), and the SPP measured on the patient's right anterior and posterior tibial artery angiosomes on his right foot changed from 51 to 72 mmHg and 14 to 11 mmHg, respectively. The patient's distal ankle PVR increased from 6 to 8 mm in the leg exposed to INP, but remained unchanged in the control leg. We also measured acute changes in transcutaneous oxygen pressure (TcPO_2_) and blood flow velocity in the dorsal pedis artery in the lower limb exposed to INP. Laser Doppler flux was measured in the pulp of both big toes during a 10‐min sequence of INP (Fig. 2).

### Patient 2

The patient was a 61‐year‐old Caucasian male with paraplegia and complete paralysis at the level of Th6‐7. He was a non‐smoker and non‐diabetic with a large deep wound on his heel, and a smaller wound at the dorsum of the foot (Table 1). The size and depth of the wounds had been unchanged for the past 2 years. His left leg was amputated several years ago above the knee after two similar wounds did not heal and frequently became infected. The patient received wound care and had been closely followed up by a wound nurse for the past 2 years.

After 8 weeks of INP‐therapy 2 h per day, the patient's SPP in the big toe pulp increased from 44 to 95 mmHg, and his ABPI increased (Table 1). The wound on the dorsum of the foot healed completely, while the size of the wound on the heel was almost healed after 8 weeks of INP treatment (Table 1 and Fig. 1).

## Discussion

In these cases involving patients with hard‐to‐heal leg and foot ulcers, we observed that INP therapy improved ulcer healing considerably. Foot perfusion improved after completion of 8 weeks of INP‐therapy (Table 1). Blood flow and wound oxygenation are key determinants of wound healing (Sen 2009; Castilla et al. 2012; Thomas 2013), we therefore measured blood flow and TcPO_2_ during an INP sequence in patient 1. In this patient, the increase in blood flow velocity and laser Doppler flux was followed by a rise in TcPO_2_ (Fig. 2).

The clinical conditions of the two patients with the lowest foot perfusion at inclusion, patient 1 and 2, improved to the extent that amputation is no longer imminent. Patient 1 stated that he now experiences no rest pain and has improved his claudication distance and his quality of life. Our findings support the results of Smyth (1969), who reported improved wound healing in patients with leg ulcers after INP‐therapy.

The mechanisms for the observed clinical effects and increased blood flow at onset of INP remain unknown (Fig. 2, upper panel). According to Poiseuille's law, flow (*Q*) between arteries and veins is proportional to the pressure gradient (Δ*P*) between the arteries (*P*_a_) and veins (*P*_v_) as follows: *Q* = Δ*P* (*P*_a_ − *P*_v_)/*R* (Resistance), where *R* depends on vessel radius, vessel length and blood viscosity. If *P*_v_ suddenly changes (decreases when applying negative pressure), flow will abruptly increase. The observed initial increased flow response to negative pressure should take place only from arteries to the veins, since veins have valves which hinder backflow. This is supported by Smyth (1969), who found that the pressure in the veins and in the deep tissues of the calf followed closely the pressure within the vacuum chamber.

After a few seconds of negative pressure, we observed a decrease in flow velocity (Fig. 2), possibly due to continued inflow from the arteries and consequently, a reduction in the pressure gradient. Repetitive flow pulses may also affect biochemical factors. Cell culture models subjected to pulsatile shear stress have been shown to increase production of prostacyclin to more than twice the rate of cells exposed to steady shear stress and 16 times greater than that of cells in stationary cultures (Frangos et al. 1985). Similar findings on shear stress and cell culture models have been found with endothelial‐derived relaxing factor (Cooke et al. 1990). Moreover, *flowmotion* has been suggested as an important factor in tissue oxygenation (Tsai and Intaglietta 1993). Fluctuations in blood flow induced by INP may contribute to an “artificial flowmotion” effect.

Lastly, two limitations should be addressed. First, this exploratory case report was not a placebo‐controlled study with a sham intervention. The patients received traditional wound care in the hospital prior to and during the intervention. This consisted of nutrition advice, wound debridement, and dressing to optimize healing. Second, the patients were not selected according to a randomized protocol. The patients were their own controls, as they had visited the wound clinic weekly for a minimum of 5 months prior to the study. Despite these methodological limitations, it is in our opinion unlikely that ulcers would heal spontaneously to the degree observed in the present report for all cases over 8 weeks.

In conclusion, the use of INP‐therapy should be investigated further as a potential non‐invasive treatment option for patients with peripheral arterial disease and hard‐to‐heal leg ulcers.

## Ethics Approval

The experimental protocol was approved by the regional ethics committee (REK Sør‐Øst 2015/1318).

## Consent for Publication

All patients provided written informed consent to participate and to publish pictures of their wounds.

## Availability of Data and Material

The datasets analyzed during this study is available from the corresponding author on reasonable request.

## Conflict of Interests

This case study was supported in part by The Norwegian Research Council and Otivio AS. ØHS is employed by Otivio with funding from the Norwegian Research Council. Otivio AS owns and has the commercial rights to the INP technology used in the study. IM is the CSO, a co‐founder and a shareholder of Otivio AS. None of the other authors have any personal conflicts of interest – financial or otherwise. The authors alone are responsible for the content and writing of the paper.
